# Early life mental health and problematic drinking in mid-adulthood: evidence from two British birth cohorts

**DOI:** 10.1007/s00127-021-02063-3

**Published:** 2021-03-25

**Authors:** Ke Ning, Praveetha Patalay, Jennifer L. Maggs, George B. Ploubidis

**Affiliations:** 1 Centre for Longitudinal Studies, UCL Social Research Institute, University College London, London, UK; 2 MRC Unit for Lifelong Health and Ageing, University College London, London, UK; 3 College of Health and Human Development, Pennsylvania State University, State College, USA

**Keywords:** Early life mental health, Adulthood problematic drinking, Developmental perspective, 1958 National Child Development Study, 1970 British birth cohort

## Abstract

**Purpose:**

Accumulating evidence suggests that externalising problems are consistently associated with alcohol use behaviours, but findings are inconsistent regarding the role of internalising problems. We investigate whether externalising and internalising problems are associated with problematic drinking in mid-adulthood, and whether potential associations are modified by age, sex and cohort.

**Methods:**

The National Child Development Study (NCDS58, *n* = 17,633) and 1970 British Cohort Study (BCS70, *n* = 17,568) recruited new-borns in Great Britain in a single week in 1958 and 1970. Mental health was assessed with the Rutter Behaviour Questionnaire at ages 7, 11, and 16 in NCDS58 and ages 5, 10 and 16 in BCS70. Problematic drinking was measured with the CAGE questionnaire at age 33 in NCDS58 and age 34 in BCS70, and the AUDIT scale at age 44/45 in NCDS58 and age 46 in BCS70. Latent scores of externalising and internalising problems were added chronologically into lagged logistic regression models.

**Results:**

Externalising and internalising problems were associated in opposite directions with problematic drinking in mid-adulthood. Externalising was a risk factor (OR [95% CI] ranging from 1.06 [1.03, 1.10] to 1.11 [1.07, 1.15] for different ages), and internalising was a protective factor (OR [95% CI] ranging from 0.95 [0.92, 0.99] to 0.90 [0.86, 0.94] for different ages). Associations between early life mental health and mid-adulthood problematic drinking did not differ by developmental timing but were stronger in males.

**Conclusion:**

Our study provides new insights on links of externalising and internalising difficulties with alcohol use and has implications for public policy in the UK.

**Supplementary Information:**

The online version contains supplementary material available at 10.1007/s00127-021-02063-3.

## Introduction

Alcohol contributes substantially to the global burden of disease, not only through alcohol use disorders (AUD) but also via other disease consequences resulting from alcohol use, such as injuries and suicide, cardiovascular disease and cancer [1]. Knowledge on the etiological mechanisms of problematic alcohol use is needed to inform the development of more effective interventions.

Theoretical and empirical research supports the idea that alcohol problems in adulthood have their roots in early life [2]. One longstanding hypothesis is that problematic drinking develops from early life externalising problems, defined broadly as aggression and disinhibited and antisocial behaviour [3, 4]. An alternative pathway involves internalising problems, which reflects difficulties with negative affect [5]. Epidemiological evidence regarding the associations between early life externalising and internalising problems and alcohol use behaviours in adulthood has been summarised in a recent systematic review [6]. In general, externalising problems in childhood and adolescence act as a risk factor for alcohol use/misuse in adulthood, while results are equivocal with respect to internalising problems in that positive [7–9], negative [10–13] and null associations [14–17] were all reported across studies.

A series of factors (developmental timing, sex, history, culture and adjustment of externalising or internalising problems accordingly) may contribute to the inconsistencies, but no definitive conclusions can be drawn as those factors were rarely examined within the same study [6]. Externalising and internalising problems are typically measured at one timepoint (mainly adolescence), which ignores their potential changes across childhood and adolescence [18–20]. Similarly, alcohol use behaviours are mainly measured during adolescence and early adulthood, the years during which drinking is typically initiated, escalates [21], and is more influenced by contextual factors (e.g. parenting and peers’ drinking behaviours) [22, 23]. In contrast, drinking in mid-adulthood stabilizes and might be less contextually influenced [24–26]. How associations between externalising and internalising problems and drinking behaviours vary across sex and historical period is also under-explored, though it is well-established that the prevalence of externalising and internalising problems and alcohol use behaviours differs between males and females [27–30] and fluctuates over historical periods [31]. Moreover, few studies examine the role of externalising and internalising problems simultaneously, which may bias each other’s relationship with alcohol outcomes [6], considering their high comorbidity across childhood and adolescence [32].

The present study extends the current literature by investigating the association of early life externalising and internalising problems with problematic drinking in mid-adulthood, and whether these associations are modified by age, sex and cohort using two successive nationally representative longitudinal British birth cohorts.

## Methods

### Sample

We used data from two British birth cohorts born 12 years apart in England, Scotland, and Wales: the 1958 National Child Development Study (NCDS58) and the 1970 British Birth Cohort (BCS70). NCDS58 recruited 17,633 babies born in one week in March 1958 with 10 waves of data collected through 2013. BCS70 recruited 17,568 babies born in one week in April 1970 with 9 waves of data through 2016. Details about both cohorts are available elsewhere [33, 34].

### Measures

Tools used to assess externalising and internalising problems, alcohol outcomes and potential confounding factors varied across waves and cohorts. To explore the potential cohort effect in the associations between externalising and internalising problems and problematic drinking, variables that were consistently collected across cohorts were retained and harmonised for analysis.

#### Early life mental health problems

Externalising and internalising problems were measured with the parent rated Rutter Behaviour Questionnaire [35]. In NCDS58, they were assessed using 14 items at ages 7 and 11, and 18 items at age 16, while the 19-item version was collected at ages 5, 10, 16 in BCS70. Exploratory factor analysis was carried out to establish items which represent externalising and internalising problems (see ESM Tables 1, 2). Four items (fights, disobedient, destructive, and irritable) were used to assess externalising problems, and four items (being worried, solitary, fearful and miserable) for internalising problems.

#### Measures of problematic drinking

The CAGE (Cut down/Annoyed/Guilty/Eye-opener) questionnaire is a four-item self-report screening instrument for detecting alcohol problems [36]. The CAGE was collected at age 33 in NCDS58 and age 34 in BCS70. A score of 2 or more indicates a propensity for AUD [36]. The AUDIT (Alcohol Use Disorder Identification Test) was developed by the World Health Organization (WHO) to identify those with hazardous and harmful patterns of alcohol consumption [37]. The full AUDIT with 10 questions assessing past-year drinking behaviours was collected at ages 44/45 in NCDS58, but only the primary-care AUDIT (AUDIT-PC) with 5 questions was collected at age 46 in BCS70 and thus was used in the current study (see ESM Table 3). For simplicity, we use age 45 to refer to this timepoint throughout the manuscript. A score of 5 or over indicated problematic drinking [38].

#### Potential confounders

Based on previous literature [39, 40], a series of potential confounding factors that were comparable across cohorts were selected and included in our models. More information regarding how the variables were derived can be found in ESM Table 4. Confounding factors include birth weight (grams), gestational age (days), maternal smoking during pregnancy, maternal age at birth (years), ever being breastfed, whether the mother stayed at school after minimum school leaving age, whether the father stayed at school after minimum school leaving age, parents’ marital status (birth, ages 5/7, 10/11 and 16), father’s social class (birth, ages 5/7, 10/11 and 16), whether parents read to child weekly (age 5/7), whether housing tenure was owned (age 5/7), household amenities (bathroom, indoor toilet, kitchen and hot water) (ages 5/7 and 16), person room ratio in the house (ages 5/7, 10/11 and 16), whether mother worked before child went to school (age 5/7), whether the mother was separated from the child for more than one month (age 5/7), parents’ interest in child’s education (age 10/11), how many times family had moved (age 5/7), body mass index (ages 10/11 and 16), cognitive ability (age 5/7 and 10/11), physical health conditions (ages 5/7 and 10/11), and bedwetting (age 5/7).

#### Statistical analysis

Item Response Theory models were applied to derive continuous latent scores at each wave in childhood and adolescence, allowing the externalising and internalising scores to be correlated [41] (see ESM Table 5). High latent scores indicate higher externalising and internalising problems, respectively.

**Fig. 1:**
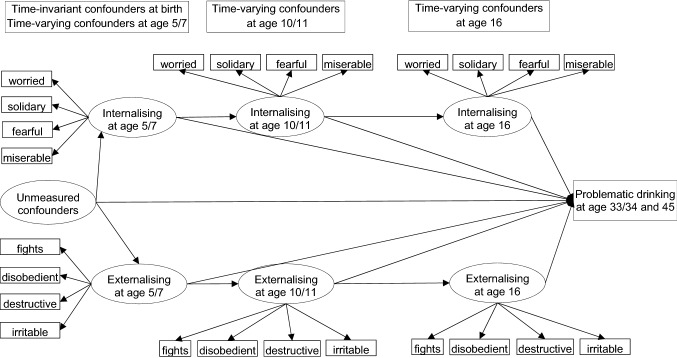
Conceptual framework. *For clarity, arrows from confounders to externalising/internalising problems at each age and problematic drinking were left out

The conceptual framework that guided our analysis is displayed in Fig. 1. Externalising and internalising problems were assumed to be associated with problematic drinking directly and indirectly through later mental health status. At the same time, time-invariant and time-varying confounding factors may bias the associations. As confounding factors such as genes or family history of alcohol problems were not collected in the cohorts, the associations after adjusting for observed confounders may still be biased by residual/unmeasured confounding. Therefore, based on the findings that high co-occurrence between externalising and internalising problems is mainly due to a common cause [32, 42–45], we argue that externalising and internalising problems at the same age should be adjusted simultaneously to reduce bias due to unmeasured confounding. For example, by adjusting externalising problems at age 5/7, the spurious association between internalising problems at age 5/7 and problematic drinking due to unmeasured common causes of externalising and internalising problems was blocked [46]. Similarly, the use of lags at ages 5/7 and 10/11 has the potential to reduce unmeasured confounding in the associations between externalising and internalising problems at ages 10/11 and 16 and problematic drinking in mid-adulthood, respectively.

Both cohorts are representative samples of births in Great Britain in 1958 and 1970. The target population for our analyses was cohort members who were still alive when the outcomes were assessed. Thus, the analytic sample size in NCDS58 is 16,600 (male = 8511, female = 8089) at age 33, and 16,336 (male = 8349, female = 7987) at age 44/45. In BCS70, it is 16,655 (male = 8601, female = 8054) at age 34, and 16,593 (male = 8586, female = 8007) at age 46. Due to attrition over more than 40 years (ESM Table 6), which is known to erode sample representativeness if left unchecked, multiple imputation (MI) was implemented. One hundred fifty imputed datasets were generated using chained equations using a two-stage calculation [47–49], and distributions of complete and imputed variables were checked [50]. Further details on the implementation of MI procedure can be found in ESM Text 1.

The analysis proceeded in two stages. In the first stage, lagged logistic regression was carried out by adding externalising scores, internalising scores and corresponding confounders chronologically to explore the direction of the association and the potential critical period of externalising and internalising problems. For example, externalising and internalising scores at age 5/7 and confounders measured at birth and age 5/7 were first included in Model 1, and then externalising and internalising scores and time-varying confounders at age 10/11 were further added in Model 2. Last, externalising and internalising scores and confounders at age 16 were added in Model 3. To investigate potential critical periods of experiencing externalising and internalising problems with respect to their associations with later problematic drinking, post hoc comparisons were conducted. For instance, comparisons were made to see whether the coefficient of externalising scores at age 5/7 in Model 1, at age 10/11 in Model 2 and at age 16 in Model 3 were equivalent to each other. Potential increases in Type I error, due to multiple comparisons, were corrected using Bonferroni corrections at a threshold of 0.017 (0.05/3) for the *P* value. In the second stage, interactions of externalising and internalising problems at each age with sex and cohort were investigated. This was achieved by adding interaction terms into the previous models. The above models are displayed in notation form in ESM Text 2.

A series of sensitivity analyses were carried out to assess the robustness of our results from various perspectives. The rationale of each analysis is articulated in ESM Text 3, and results are presented and discussed where appropriate.

## Results

As shown in Table 1, at ages 5/7 and 10, level of externalising problems was higher in males compared to that in females, and level of internalising problems was similar across sex. However, at age 16, levels of externalising and internalising problems tended to be higher in females than those in males. Prevalence of problematic drinking, though assessed using different scales, was about 2 times higher in males compared to that in females across cohorts at ages 33/34 and 45.

**Table 1:** Descriptive statistics for externalising and internalising score and problematic drinking across sex and cohorts

	NCDS58				BCS70			
	Male		Female		Male		Female	
	Mean ± SD							
Externalising score at age 5/7	7319	0.16 ± 1.04	6933	− 0.17 ± 1.05	6698	0.18 ± 1.18	6232	− 0.20 ± 1.11
Internalising score at age 5/7	7319	− 0.00 ± 1.26	6933	+ 0.00 ± 1.28	6698	0.03 ± 1.10	6232	− 0.03 ± 1.14
Externalising score at age 10/11	6821	0.17 ± 1.11	6480	− 0.18 ± 1.08	6750	0.18 ± 1.45	6373	− 0.19 ± 1.28
Internalising score at age 10/11	6821	0.01 ± 1.34	6480	− 0.02 ± 1.37	6750	0.03 ± 1.51	6373	− 0.04 ± 1.56
Externalising score at age 16	5692	− 0.09 ± 1.58	5432	0.10 ± 1.60	4123	− 0.07 ± 1.85	4320	0.07 ± 1.82
Internalising score at age 16	5692	− 0.14 ± 1.15	5432	0.15 ± 1.26	4123	− 0.14 ± 1.38	4320	0.13 ± 1.51

	*N* (%)							
CAGE at age 33/34	5359	924 (17.24%)	5543	437 (7.88%)	4414	1031 (23.36%)	4779	650 (13.60%)
AUDIT-PC at age 45	4439	1933 (43.55%)	4514	901 (19.96%)	3973	1217 (30.63%)	4292	719 (16.75%)

Externalising and internalising problems at each age in childhood and adolescence were associated in opposite directions with problematic drinking, and these associations persisted across mid-adulthood (see Table 2). Externalising problems acted as a risk factor for problematic drinking in mid-adulthood, while internalising problems served as a protective factor. The size of the *E* value ranged from 1.20 to 1.29, indicating that an unmeasured confounding factor would still need to be associated with externalising or internalising problems and problematic drinking at a risk ratio of 1.20–1.29 to nullify the observed association to 1. The strength needed to render the confidence interval to include 1 was smaller (1.08–1.22).

**Table 2:** Associations between externalising and internalising problems and problematic drinking at age 33/34 and age 45 in two British birth cohorts

	Model 1	*E* value^a^	Model 2	*E* value	Model 3	*E* value
PD (CAGE) at age 33/34						
EXT at age 5/7	1.10 (1.05, 1.15)***	1.28 (1.18)	1.07 (1.02, 1.12)**	NA	1.05 (1.00, 1.10)	NA
INT at age 5/7	0.96 (0.92, 1.00)	1.17 (1.00)	0.97 (0.93, 1.02)	NA	0.98 (0.93, 1.02)	NA
EXT at age 10/11			1.09 (1.04, 1.15)***	1.26 (1.16)	1.05 (1.00, 1.11)*	NA
INT at age 10/11			0.95 (0.92, 0.99)*	1.19 (1.08)	0.97 (0.93, 1.01)	NA
EXT at age 16					1.11 (1.06, 1.16)***	1.29 (1.20)
INT at age 16					0.93 (0.87, 0.99)*	1.23 (1.08)
*N*	33,255		33,255		33,255	
PD (AUDIT-PC) at age 45						
EXT at age 5/7	1.06 (1.03, 1.10)***	1.20 (1.14)	1.05 (1.01, 1.09)*	NA	1.03 (0.99, 1.08)	NA
INT at age 5/7	0.94 (0.91, 0.98)***	1.21 (1.11)	0.96 (0.93, 1.00)*	NA	0.97 (0.94, 1.01)	NA
EXT at age 10/11			1.07 (1.03, 1.11)**	1.22 (1.14)	1.03 (0.99, 1.08)	NA
INT at age 10/11			0.94 (0.91, 0.97)***	1.21 (1.14)	0.96 (0.93, 0.99)*	NA
EXT at age 16					1.11 (1.07, 1.15)***	1.29 (1.22)
INT at age 16					0.90 (0.86, 0.94)***	1.29 (1.21)
*N*	32,929		32,929		32,929	

Confounding factors were added chronologically as described in the method section. Logistic regression was run, and thus results are reported as OR (95%). Scale of latent score for externalising and internalising problems was constrained by fixing the factor loading of “worried” and “irritable” to 1 to ensure the comparability across ages and cohorts; coefficients of standardised latent score can be found in ESM Table 7

*EXT* externalising problems, *INT* internalising problems, *PD * problematic drinking

*p < 0.05, **p < 0.01, ***p < 0.001

^a^NA refers to not applicable. The value outside the bracket is *E* value for the point estimate, and the value in the bracket is *E* value for the limit of the confidence interval closest to the null (the strength needed to move the confidence interval to include 1)

Post hoc comparisons indicate that the strength of the associations (absolute value of the coefficients) with problematic drinking did not differ between externalising and internalising problems, and that the strength of the associations between externalising or internalising problems measured at age 5/7, age 10/11 and age 16 and problematic drinking in adulthood did not differ by age.

**Fig. 2:**
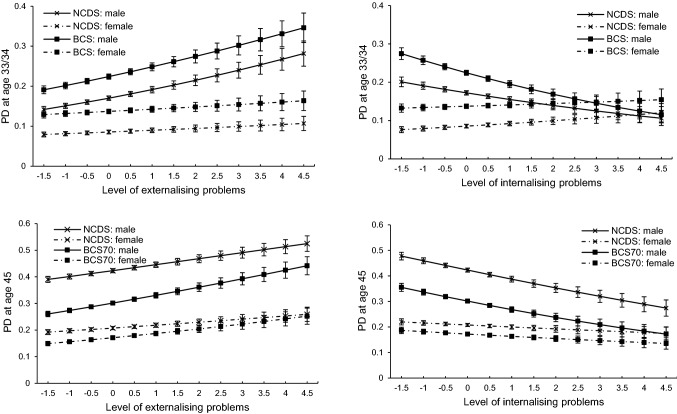
Probability of problematic drinking (PD) at different level of externalising and internalising problems at age 16

Interactions of externalising and internalising problems with sex predicting the probability of problematic drinking were mainly detected at age 16. These are plotted in Fig. 2 with more detail in ESM Table 8. We observed an interaction between externalising problems at age 16 and sex predicting problematic drinking at age 33/34 (*p* = 0.028), with the association between externalising problems and problematic drinking at age 33/34 being observed only in males. An interaction between internalising problems and sex was detected at both age 33/34 (*p* < 0.001) and age 45 (*p* = 0.026), with the association being stronger in males.

We did not observe interactions between externalising or internalising problems and cohort regarding their associations with problematic drinking at any age (ESM Table 8).

## Discussion

By analysing data from two population-based prospective British birth cohorts born 12 years apart, we found that externalising problems across childhood and adolescence acted as a risk factor for problematic drinking in adulthood. In contrast, a negative association between internalising problems and problematic drinking was observed, which indicates that those experiencing internalising symptoms in childhood or adolescence were less likely to exhibit problematic drinking in mid-adulthood. We did not detect a critical period of experiencing externalising and internalising problems between early childhood and mid-adolescence regarding their associations with problematic drinking. Strength of the associations did not differ significantly across cohorts but were stronger in males.

Existing evidence is quite inconsistent regarding the association between early life internalising problems and alcohol use behaviours in adulthood [51, 52]. This may be due to methodological differences, since studies that found positive associations either measured alcohol outcomes in adolescence or early adulthood (from age 16 to age 25) or were conducted in American or Australian settings, and crucially did not adjust for externalising problems [53, 54]. By comparison, studies reporting negative associations either measured alcohol outcomes at mid/late adulthood (age 26 onwards) and adjusted for externalising problems [10, 12, 55] or were conducted in the UK [10, 12]. Thus, the life course stage at which alcohol behaviour was measured, whether externalising problems were adjusted for, as well as the cultural context might explain part of the divergence among studies.

The association between early life internalising problems and later alcohol use behaviours may vary across the life course, as determinants and patterns of drinking typically change with development. Adolescence is a period of dramatic changes physically, psychologically and socially; drinking in this period is strongly socially-driven, greatly affected by social norms, peer influences and parents’ monitoring [22, 56]. For many, early adulthood is characterised by a series of role transitions that coincide with normative but not universal reductions in alcohol consumption [57, 58]. In mid-adulthood, lifestyle behaviours often have solidified into more regular patterns [26], and may reflect longer-term origins back in childhood and adolescence [59–61]. Here, adults who had experienced more internalising problems early in life were less likely to report problematic drinking in mid/late adulthood.

Furthermore, the association between early life internalising problems and alcohol use behaviours in adulthood may vary across populations. The proposed internalising pathway to alcohol use/disorder suggests that people with internalising problems use alcohol to self-medicate or gain acceptance by peers [5]. However, this may not be the primary mechanism of action in the British context, where alcohol has been used as a way of socializing at least since its popularity increased after World War II [62]. Children or adolescent with internalising problems (measured via items capturing solitary, fearful of new things, worried about many things and miserable/tearful) may experience reduced exposure to alcohol because they spent less time at pubs, bars and clubs. This aligns with the hypothesis that internalising tendencies towards social withdrawal and fear of negative consequences may decrease the risk of problematic drinking through reducing one’s exposure to alcohol use [63, 64]. Our results indicate that the protective effect of early life internalising problems persists into mid-adulthood in the UK context, and are consistent with another UK-based population study [12].

**Fig. 3:**
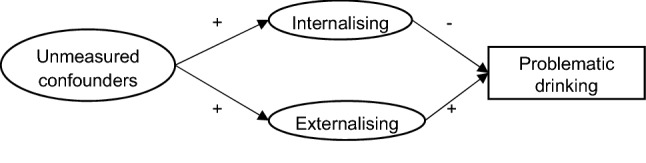
DAG illustration of the suppressing effect between externalising and internalising problems

Our results support the possibility that not adjusting for externalising problems could introduce residual confounding in the association between internalising problems and alcohol outcomes. The observed opposite direction of the associations of externalising and internalising problems with alcohol outcomes has also been found in previous studies where externalising and internalising problems were adjusted for simultaneously [10, 13, 65–68]. In our study, we observed systematic attenuation of the association when externalising and internalising problems were added into the model separately (see ESM Tables 9, 10 and Table 2). To illustrate with a Directed Acyclic Graph in Fig. 3 [46, 69], when externalising problems were not adjusted, the association between internalising and problematic drinking was a combination of two paths. One represented the path of interest (internalising problems → problematic drinking) and the other carried a spurious association (internalising problems ← unmeasured confounders → externalising problems → problematic drinking) which was positive. Thus, when externalising problems were not adjusted, the true association of interest which was negative could be attenuated, cancelled out or even reversed depending on the strength of the association between externalising problems and alcohol outcomes in the population. When a general psychological factor that captured the common variance of externalising and internalising problems was employed as an exposure, we found that the association between the psychopathological factor and problematic drinking was the average of the association of externalising problems with problematic drinking and that of internalising problems with problematic drinking (ESM Table 11). In addition, we did not observe large standard errors or variance inflation factors [70]. Therefore, the observed opposite associations between externalising and internalising problems with problematic drinking should be seen as evidence of a suppressing effect rather than a consequence of multicollinearity.

Results from post hoc comparisons did not identify any critical periods in childhood or adolescence when experiencing mental health problems showed a stronger or weaker association with problematic drinking in adulthood. Few studies have had sufficient longitudinal data to explore the developmental timing of externalising or internalising behaviours and their potential impact on alcohol use behaviours in mid-adulthood. Individuals with externalising problems may actively self-select into environments and peer settings where alcohol use is common, consistent with their dispositions, and they may be reinforced reciprocally by these environments [71]. Person-based analysis that derived trajectories of externalising problems, showed that externalising problems in late adolescence may be a key dimension: compared to those with consistently low level of externalising problems, those with either early-onset-persistent or adolescent-onset externalising problems had worse outcomes, while childhood-limited externalising problems were not associated with later alcohol use behaviours [72–77]. In this context, the detection of a critical period may depend on the proportion of individuals with childhood-limited and adolescent-onset externalising problems in the population. By adjusting latent scores of previous externalising and internalising problems in the model, our results avoid the influence of the constitution of heterogeneous trajectories in the population and indicate that there were no critical periods of mental health problems between childhood and adolescence regarding the strength of their relationship with problematic drinking in the British population. However, this conflicting implication between results of our study and those of person-based analysis warrants more exploration, and future research would offer new insights if both approaches could be carried out within one study.

Another finding worth our attention is how the associations between externalising and internalising problems and problematic drinking differed significantly across sex but not cohorts. Born 12 years apart, participants from NCDS58 and BCS70 were raised during a time of shifting social environments towards alcohol [78, 79]. Though the prevalence of problematic drinking at different ages differed significantly across cohorts, the strength of the associations between externalising and internalising problems and problematic drinking generally did not differ, which is consistent with previous findings [31]. At least for these two generations born in 1958 and 1970, the observed associations between early life mental health and later problematic drinking reflect general developmental processes, and there is no evidence that their association reflects historical changes in patterns and culture of alcohol use [31]. However, we did see suggestive evidence for stronger links between externalising problems in childhood and adolescence and problematic drinking in midlife in the later-born BCS70 cohort than those in the NCDS58 cohort. That is, the interaction terms between externalising scores and cohort (NCDS58 as reference) with problematic drinking at age 45 tended to be positive (ESM Table 8), and the curve between the externalising score and the probability of problematic drinking at age 45 was steeper in BCS70 compared to that in NCDS58 (Fig. 2). These differences, if sustained in the long run in more recently born cohorts, will lead to a stronger association between externalising problems and problematic drinking. Modification by sex in the associations of externalising and internalising problems with problematic drinking was found with the associations being stronger in males, especially for internalising problems. Traditionally and even today in the UK, males consumed alcohol more frequently in pubs [62, 80]. As earlier life internalising problems in our study were assessed using indicators such as being “fearful” and “solitary”, this may help explain the stronger association among males. The majority of prior studies examining interactions between mental health problems and sex predicting alcohol use in adulthood have found none [7, 8, 15, 73, 81–87], or, have observed inconsistent results [55, 88–90]. Noticeably, for generations born around or after 1980s in the UK, women have been shown to consume alcohol similarly to their male counterparts [91]. Thus, we could not rule out the possibility that variation of the association across sex reported here would disappear for recent generations in the UK.

### Policy implications

If causal, our findings have implications for public policy. First, externalising problems instead of internalising problems might be a better target for early life intervention to alleviate alcohol burden in adulthood in the UK context. Though the high co-occurrence between externalising and internalising problems is well-established [92, 93], most existing intervention programmes shown to be effective target them separately [94]. Second, strategies of promoting sensible drinking should be incorporated when targeting internalising problems to tackle their detrimental effects on other aspects of life (e.g. adult mental health, psychosocial outcomes) [95, 96]. One should be vigilant of the potential subsequent alcohol burden after the intervention. Third, more evidence is warranted to examine whether the externalising pathway is stronger in males in generations born after 1970 and to explain the discrepancies regarding possible critical periods of mental health from both person-based and variable-based perspectives. Such evidence will have further implications on who (males versus females) and when (at which age) should be targeted in more recent generations to alleviate their potential alcohol burden.

### Strengths and limitations

Strengths of our study include the availability of two well-characterised prospective national birth cohorts with repeated measures of mental health that span childhood and adolescence and measures of problematic drinking in early adulthood and midlife. This adds to the scarce evidence on how far into adulthood the pathway from early mental health to alcohol use persists. Furthermore, considering the high rate of co-occurrence between externalising and internalising problems in childhood and adolescence [32, 42, 97], the association of externalising and internalising problems with problematic drinking was investigated both simultaneously and separately to investigate their potential suppressing effect on each other, which has been ignored in most previous studies. Various forms of sensitivity analyses supported the robustness of our findings. Our analytic approach aimed to reduce bias from, arguably, its three major sources in observational studies: measurement error (ESM Text4 and ESM Tables 12–17), residual confounding (Table2 and ESM Table 9, 10, 18), and missing data related selection bias (ESM Table 19) [98].

Several limitations should also be considered when interpreting our findings. As in any other longitudinal survey, selective attrition is unavoidable. In this instance, about 60% of the original sample was retained by age 46 in both cohorts. By making use of the abundant information collected in both longitudinal studies [99], we imputed 150 datasets and carried out sensitivity analysis with only cases with complete outcome data. Though results showed that our results were quite robust to attrition, selection bias cannot be ruled out. Furthermore, our measurement may not reflect the whole range of externalising and internalising problems. Only four items were retained to assess externalising and internalising problems. Though the latent scores derived utilising four items are highly correlated with the latent scores derived utilising all relevant items in the Rutter scales (*r* > 0.95 for NCDS58, *r* > 0.92 for BCS70), externalising and internalising problems in our study should be interpreted with caution, especially when comparing the results with other studies. Third, the short version of AUDIT used in our study increases the rate of false positives in detecting problematic drinking (Table 1 and ESM Tables 14, 15) and biases our results towards the null. This was observed in sensitivity analysis when the full-AUDIT scale in NCDS58 was utilised (ESM Table 16). Fourth, despite the relatively rich set of confounders that were included in the models, other potential confounders (e.g. family history of alcoholism, parenting strategy, peers’ drinking behaviours) were not adjusted in our study due to their unavailability. Adjusting highly correlated externalising and internalising problems simultaneously and their lags has the potential of blocking these potential confounders at least partially (ESM Tables 9, 10, 18). In addition, the *E* value, which evaluates the minimum strength—on the risk ratio scale—that an unmeasured confounder would need to have with both exposure and outcome to fully explain away the observed association (conditional on the measured confounding factors), was calculated [100]. However, the size of the *E* value in our study (Table 2) could be interpreted from two perspectives. On one hand, the size of the *E* value (1.20–1.29) indicates that the association of the unmeasured confounder with problematic drinking, conditional on all the covariates included in the model, would need to be stronger than the observed associations of externalising problems with problematic drinking (1.06–1.11) in our study. Thus, it could be argued that the observed associations were less likely to be fully nullified by unmeasured confounding factors, as the externalising pathway is, up to now, shown to be the most robust pathway [101]. On the other hand, the size of the E-value could also be seen to indicate that the observed associations were still susceptible to unmeasured confounding factors, including genetics [102, 103], parental psychopathology and substance use and peers’ drinking behaviours [104]. However, the lack of studies examining the strength of those potential confounders with problematic drinking into mid-adulthood while taking externalising and internalising problems into account constrains further speculations. Future studies would offer more insights if they could examine whether genetics and familial and social risk factors act as potential confounding factors for the pathway from externalising problems to problematic drinking or act as upstream factors which are linked to problematic drinking only through externalising problems.

## Conclusion

By utilising two British birth cohorts, we found that externalising problems in childhood and adolescence were positively associated with problematic drinking across mid-adulthood while internalising problems were negatively associated with problematic drinking, and the results were robust in a series of sensitivity analyses. We also found no evidence for a particular critical period for experiencing externalising and internalising problems in childhood or adolescence that was uniquely important as predictors of problematic drinking in mid-adulthood. The stability of these associations across two UK cohorts born 12 years apart indicates the developmental nature of the association between externalising and internalising problems and problematic drinking. More evidence is needed to examine whether the detected sex differences in the associations persist for more recent generations. Our study provides new insights on links of externalising and internalising problems with alcohol use in the UK. If causal, our findings could inform design of early life interventions to reduce alcohol burden in adulthood.

## Supplementary Information

Below is the link to the electronic supplementary material.

Supplementary file1 (DOCX 114 KB)

## Data Availability

Available upon request.

## Code availability

Available upon request.
