# Supplementary material for: Early life mental health and problematic drinking in mid-adulthood: evidence from two British birth cohorts
Source: Soc Psychiatry Psychiatr Epidemiol. 2021 Mar 25;56(10):1847–58. doi: 10.1007/s00127-021-02063-3 (PMC8429378; doi:10.1007/s00127-021-02063-3)
Supplement: Supplementary file 1 — Supplementary file1 (DOCX 114 KB) [file 127_2021_2063_MOESM1_ESM.docx]

**Early life mental health and problematic drinking in mid-adulthood: Evidence from two British birth cohorts**

Ke NING^1*^, Praveetha Patalay^1,2^, Jennifer Maggs^3^, George B. Ploubidis^1^

^1^ Centre for Longitudinal Studies, Department of Social Science, University College of London, London, UK

^2^ MRC Unit for Lifelong Health and Ageing, University College of London, London, UK

^3^ College of Health and Human Development, Pennsylvania State University, USA

ESM Table 1. Exploratory factor analysis for Rutter Behaviour Questionnaire in NCDS58

ESM Table 2. Exploratory factor analysis for Rutter Behaviour Questionnaire in BCS70

ESM Table 3. CAGE and AUDIT questions in NCDS58 and BCS70 across waves

ESM Table 4. Potential confounding factors

ESM Table 5. Factor loadings for latent externalising and internalising score in NCDS58 and BCS70

ESM Table 6. Missingness in NCDS58 (upper half) and BCS70 (lower half) across waves

ESM Text 1. Multiple imputation procedure

ESM Text 2. Formula illustration of statistical models

ESM Table 7. Interaction between externalising and internalising behaviour and sex/cohort regarding their association with problematic drinking at age 33/34 and age 45 in two British birth cohorts

ESM Table 8. Association between early life externalising and internalising problems and problematic drinking at age 33/34 in two British birth cohorts (not adjusting each other)

ESM Table 9. Association between early life externalising and internalising problems and problematic drinking at age 45 in two British birth cohorts (not adjusting each other)

ESM Table 10. Distribution of weekly alcohol units and prevalence of problematic drinking across mid-adulthood in NCDS58

ESM Table 11. Distribution of weekly alcohol units and prevalence of problematic drinking across mid-adulthood in BCS70

ESM Table 12. Variables collected with information about various drinking behaviours in NCDS58 and BCS70

ESM Text 3. Conversion to UK units

ESM Table 13. Association between externalising and internalising behaviour and weekly alcohol units at age 33/34 in two British birth cohort

ESM Table 14. Association between early life externalising and internalising problems and problematic drinking (PD) at age 45 measured using different AUDIT scale in NCDS58

ESM Table 15. Association between sum score of early life externalising and internalising problems and problematic drinking (PD) at age 33/34 and age 45 in two British birth cohorts

ESM Table 16. Comparison of the coefficients before and after adjusting previous externalising and internalising problems

ESM Table 17. Association between early life externalising and internalising problems and problematic drinking (PD) at age 33/34 and age 45 in two British birth cohorts

ESM Table 1. Exploratory factor analysis for Rutter Behaviour Questionnaire in NCDS58

|  |  | Age 7 |  |  | Age 11 |  |  | Age 16 |  |
| --- | --- | --- | --- | --- | --- | --- | --- | --- | --- |
|  | Conduct problems | Internalising problems | ADHD | Conduct problems | Internalising problems | ADHD | Conduct problems | Internalising problems | ADHD |
| Poor concentration | 0.44 | -- | 0.43 | 0.39 | -- | 0.45 | 0.35 | -- | 0.64 |
| Fidgety | 0.45 | -- | 0.44 | 0.41 | -- | 0.49 | -- | -- | 0.82 |
| Sucks thumb | -- | -- | -- | -- | -- | -- | -- | -- | -- |
| Twitches/tics | -- | -- | -- | -- | -- | -- | -- | -- | 0.36 |
| Bites nails | -- | -- | -- | -- | -- | -- | -- | -- | -- |
| Restless | NC | NC | NC | NC | NC | NC | -- | -- | 0.82 |
| Solitary | -- | -- | -- | -- | -- | -- | -- | 0.41 | -- |
| Miserable | 0.40 | 0.42 | -- | 0.44 | 0.39 | -- | 0.44 | 0.50 | -- |
| Worries | -- | 0.67 | -- | -- | 0.67 | -- | -- | 0.66 | -- |
| Fearful | -- | 0.55 | -- | -- | 0.57 | -- | -- | 0.60 | -- |
| Fussy | NC | NC | NC | NC | NC | NC | -- | 0.36 | -- |
| Destructive | 0.55 | -- | -- | 0.62 | -- | -- | 0.66 | -- | 0.37 |
| Irritable | 0.58 | -- | -- | 0.58 | -- | -- | 0.56 | -- | -- |
| Fights | 0.55 | -- | -- | 0.57 | -- | -- | 0.76 | -- | -- |
| Disobedient | 0.66 | -- | -- | 0.63 | -- | -- | 0.70 | -- | -- |
| Not liked | NC | NC | NC | NC | NC | NC | 0.48 | 0.37 | -- |
| Steals | NC | NC | NC | NC | NC | NC | NC | NC | NC |
| Lies | NC | NC | NC | NC | NC | NC | 0.68 | -- | -- |
| Bullies | NC | NC | NC | NC | NC | NC | 0.75 | -- | -- |
| Bullied | -- | 0.41 | -- | -- | 0.39 | -- | NC | NC | NC |

*NC=Not collected. Factor loadings below 0.3 are not displayed.

ESM Table 2. Exploratory factor analysis for Rutter Behaviour Questionnaire in BCS70

|  |  | wave1 |  |  |  | wave2 |  |  |  | wave3 |  |
| --- | --- | --- | --- | --- | --- | --- | --- | --- | --- | --- | --- |
|  | Conduct problems | ADHD | Internalising problems |  | Conduct problems | ADHD | Internalising problems |  | Conduct problems | Internalising problems | ADHD |
| Poor concentration | 0.33 | 0.57 | -- |  | 0.36 | 0.57 | -- |  | 0.49 | -- | 0.51 |
| Fidgety | -- | 0.69 | -- |  | -- | 0.72 | -- |  | 0.30 | -- | 0.74 |
| Sucks thumb | -- | -- | -- |  | -- | -- | -- |  | -- | -- | -- |
| Twitches/tics | -- | -- | -- |  | 0.40 | -- | -- |  | -- | -- | 0.42 |
| Bites nails | -- | -- | -- |  | -- | -- | -- |  | -- | -- | -- |
| Restless | -- | 0.70 | -- |  | -- | 0.78 | -- |  | 0.30 | -- | 0.67 |
| Solitary | -- | -- | 0.38 |  | -- | -- | 0.44 |  | -- | 0.44 | -- |
| Miserable | -- | -- | 0.51 |  | 0.40 | -- | 0.51 |  | 0.47 | 0.58 | -- |
| Worries | -- | -- | 0.66 |  | -- | -- | 0.72 |  | -- | 0.72 | -- |
| Fearful | -- | -- | 0.53 |  | -- | -- | 0.65 |  | -- | 0.61 | -- |
| Fussy | -- | -- | 0.46 |  | -- | -- | 0.45 |  | -- | 0.45 | -- |
| Destructive | 0.60 | 0.33 | -- |  | 0.68 | 0.35 | -- |  | 0.73 | -- | 0.31 |
| Irritable | 0.42 | 0.30 | 0.32 |  | 0.40 | 0.38 | 0.36 |  | 0.55 | 0.43 | -- |
| Fights | 0.69 | -- | -- |  | 0.62 | 0.31 | -- |  | 0.74 | -- | -- |
| Disobedient | 0.58 | 0.38 | -- |  | 0.59 | 0.43 | -- |  | 0.76 | -- | -- |
| Not liked | 0.41 | -- | 0.31 |  | 0.40 | -- | 0.33 |  | 0.50 | 0.35 | -- |
| Steals | 0.62 | -- | -- |  | 0.80 | -- | -- |  | 0.79 | -- | -- |
| Lies | 0.60 | -- | -- |  | 0.75 | -- | -- |  | 0.81 | -- | -- |
| Bullies | 0.68 | -- | -- |  | 0.69 | -- | -- |  | 0.78 | -- | -- |

*Factor loadings below 0.3 are not displayed.

ESM Table 3. CAGE and AUDIT questions in NCDS58 and BCS70 across waves

|  |  | NCDS58 | | | |  | BCS70 | | | |
| --- | --- | --- | --- | --- | --- | --- | --- | --- | --- | --- |
|  | Year | 1991 | 2000 | 2002/2004 | 2008 |  | 2000 | 2004 | 2012 | 2016 |
| Scale | Age | 33 | 42 | 44-46 | 50 |  | 30 | 34 | 42 | 46 |
| CAGE | Have you ever felt you needed to Cut down on your drinking? | √ | √ |  |  |  | √ | √ |  |  |
|  | Have people Annoyed you by criticizing your drinking? | √ | √ |  |  |  | √ | √ |  |  |
|  | Have you ever felt Guilty about drinking? | √ | √ |  |  |  | √ | √ |  |  |
|  | Have you ever felt you needed a drink first thing in the morning (Eye-opener) to steady your nerves or to get rid of a hangover? | √ | √ |  |  |  | √ | √ |  |  |
| AUDIT | How often do you have a drink containing alcohol? |  |  | √ | √ |  |  |  | √ | √ |
|  | How many drinks containing alcohol do you have on a typical day when you are drinking? |  |  | √ | √ |  |  |  | √ | √ |
|  | How often do you have six or more drinks on one occasion |  |  | √ | √ |  |  |  |  |  |
|  | How often during the last year have you found that you were not able to stop drinking once you had started |  |  | √ | √ |  |  |  | √ | √ |
|  | How often during the last year have you failed to do what was normally expected from you because of drinking? |  |  | √ | √ |  |  |  | √ | √ |
|  | How often during the last year have you needed a first drink in the morning to get yourself going after a heavy drinking session? |  |  | √ | √ |  |  |  |  |  |
|  | How often during the last year have you had a feeling of guilt or remorse after drinking? |  |  | √ | √ |  |  |  |  |  |
|  | How often during the last year have you been unable to remember what happened the night before because you had been drinking? |  |  | √ | √ |  |  |  |  |  |
|  | Have you or someone else been injured as a result of your drinking? |  |  | √ | √ |  |  |  |  |  |
|  | Has a relative or friend or a doctor or other health worker been concerned about your drinking or suggested you cut down? |  |  | √ | √ |  |  |  | √ | √ |

ESM Table 4. Potential confounding factors

| Variable | Age | Description |
| --- | --- | --- |
| Maternal age at birth | 0 in both NCDS58 and BCS70 | In both NCDS58 and BCS70, maternal age at birth was collected |
| Breast-feeding or not | 0 in both NCDS58 and BCS70 | In both NCDS58 and BCS70, data were collected on whether the mother breastfed the infant or not |
| Pregnancy smoke | 0 in both NCDS58 and BCS70 | In NCDS58, mothers were asked how much they smoked per day before pregnancy and whether this behaviour changed during pregnancy. In BCS70, detailed question on whether the mother smoked during pregnancy and how much they smoked per day was collected. Binary variable indicating whether the mother smoked more than 1 cigarette on a daily basis was created. |
| Gestational days | 0 in both NCDS58 and BCS70 | Gestational days in both cohorts |
| Birthweight | 0 in both NCDS58 and BCS70 | In NCDS58, birthweight was recorded in ounces and transformed into grams; In BCS70, birthweight was recorded in grams |
| Social Class | 0, 7, 11, 16 in NCDS58; 0, 5, 10, 16 in BCS70 | Occupation of the father was coded according to the Registrar General's classification. Participant's current or most recent jobs were classified as: I (professional), II (managerial and technical), III (skilled non-manual/manual), IV (partly-skilled), and V (unskilled). Family with no male head or unemployed were coded as missing. |
| Parental education | 0 in both NCDS58 and BCS70 | In NCDS58, questions were asked on whether the mother and father continued to stay at school after minimum school leaving age (Yes/No); In BCS70, questions were collected on age when the mother and father left school. Because minimum school leaving age was raised to 16 in 1974, a cut-off point of age was applied. |
| Marital status | 0, 7, 11, 16 in NCDS58; 0, 5, 10, 16 in BCS70 | In both NCDS58 and BCS70, marital status of the mother was recorded. Married/stable union/twice married were coded into one category, and unmarried/widow/separated/divorce were coded into another category. Marital status at later ages mainly reflects whether the participants lived together with their natural parents at the moment. In NCDS58, when participants became 33 years old, they were asked to recall whether their parents had ever permanently separated or divorced, if so, how old they were when it happened. Marital status at age 7, 11 and 16 was then derived based on the above two questions. In BCS70, when participants were age 16, they were also asked to recall whether they lived with their natural parents at age 5, 10 and 16. |
| Read to child every week | 7 in NCDS58 and 5 in BCS70 | In NCDS58, how often mother and father read to the child was recorded (every week/occasionally/hardly ever). Those family where either mother or father read to the child every week were coded into one category (read to every week) and the rest into another category. In BCS70, questions on who read to the child most often and how many days the child was read to last week were collected. Those family where mother or father read to the child most often and days read to last week were greater than zero were coded into one category (read to every week) and the rest into another category. |
| House tenure | 7 in NCDS58 and 5 in BCS70 | In NCDS58, information on tenure of the accommodation was collected (owner occupied, council rented, private rented, rent free and other). In BCS70, similar information was also recorded (owned outright, being bought, council rented, private rented but unfurnished, private rented but furnished, tied to occupation and other). Thus, a binary variable indicates whether the accommodation is tenure was created. |
| House amenity access | 7 & 16 in NCDS58 and 5 & 16 in BCS70 | In NCDS58, access (sole use, shared use, no facility) to household amenities (bathroom, indoor WC, outdoor WC, kitchen, hot water) was collected at age 7, 11, 16. In BCS70, access to household amenities (bathroom, indoor WC, outdoor WC, hot water, kitchen) at age 5 and access to household amenities (bathroom, indoor WC, hot water, kitchen) at age 16 were collected. Thus, binary variable indicating whether household had sole use to bathroom, indoor WC, kitchen and hot water was created at age 7&16 in NCDS58 and at age 5&16 in BCS70. |
| Crowdness in the household | 7, 11, 16 in NCDS58; 5, 10, 16 in BCS70 | In NCDS58 and BCS70, number of people in the household and number of rooms (exclude kitchen, toilet, bathroom and any room used for business/trade) in the household was asked. Number of persons per room (up to 1, over 1 to 1.5, over 1.5) was then created. In BCS70, at age 16, total number of people in the household was calculated using number of people younger than the target, exactly same age with the target, older than the target but below age 21, older than 21, and number of room in the household was calculated using number of bedroom and number of other rooms. |
| Whether mother worked before child went to school | 7 in NCDS58 and 5 in BCS70 | In NCDS58, information on whether mother went to paid work outside the home since child's birth and before child started school (Part-time/temporary, full-time, has not worked); In BCS70, has mother had a regular full-time or part-time job out of the home since the time of N's birth which she subsequently gave up (full-time job, part-time job, both full and part-time job, never had a job out of the home, other). A binary variable indicating whether the mother had worked outside the home was created. |
| Separated with the child for more than one month | 7 in NCDS58 and 5 in BCS70 | In both NCDS58 and BCS70, question on the mother and the child were separated for more than one month was asked. |
| Interested in Education | 11 in NCDS58 and 10 in BCS70 | In NCDS58, level of mother and father's interest (over-concerned, very interested, some interest, little interest) in child's education was asked at age 7, 11 & 16. Same information was collected at age 10 in BCS70. Thus, those family where either mother or father showed great interest (over-concerned, very interested) in the child's education was coded as very interested, and those where both of the parents showed little interest were coded as little interest and the rest were coded as some interest. Variable was created for age 11 in NCDS58 and for age 10 in BCS70. |
| Times of family moves | 7 in NCDS58 and 5 in BCS70 | In both NCDS58 and BCS70, how many times has the family moved houses since child's birth. A three-category variable was created (no moves, 1-2 moves, 3+ moves) |
| BMI | 11 & 16 in NCDS58 and 10 & 16 in BCS70 | Height and weight were measured by trained medical personnel using standard protocols at age 7, 11 & 16 in NCDS58 and at age 10 & 16 in BCS70. Thus, BMI was created for both cohorts and were harmonised by the CLOSER consortium to facilitate comparisons across cohorts. |
| Cognitive ability | 7, 11 in NCDS58 and 5, 10 in BCS70 | In NCDS58, copy and design test, drawing a man, Southgate Group Reading Test, Problem arithmetic test was carried out at age 7; Copy and design, reading comprehension, mathematics test and general ability test was carried out at age 11; Reading comprehension and mathematic comprehension was done at age 16. In BCS70, copy and design test, drawing a man, English Picture Vocabulary test, Profile test, Reading test was done at age 5; tests carried out at age 10 includes Shortened Edinburgh Reading test, British Ability Scales, the CHES Pictorial Language Comprehension test, the CHES Friendly math test, Social Judgement scale, Diagnostic Measures; only Vocabulary and spelling test was done at age 16. Thus, for cross-cohort comparison, copy and design test and drawing a man were used at age 7 in NCDS58 and at age 5 in BCS70 (variables were transformed to be on the same scale); general ability test which contains verbal and non-verbal domain at age 11 in NCDS58 and British Ability Scales which contains four subsets (word definition and word similarity for verbal skills, recall of digits and matrices for non-verbal skills) at age 10 in BCS70 were used. For both cohorts, Principle Component Analysis was conducted to extract the common variance across verbal and non-verbal scales. Specifically, for BCS70, items within each subset were first summed up, and PCA was applied to extract score for verbal and non-verbal scales separately. Then a general ability score was calculated by applying PCA to verbal and non-verbal scales. All variables were standardised to a mean of 0 and a SD of 1. |
| Medical conditions | 7, 11 in NCDS58 and 5, 10 in BCS70 | Whether a series of comparable medical conditions (eczema, hay fever and sneezing, ear discharge, sore throats, bronchitis, pneumonia, hearing difficulty) had ever happened were both collected at age 7 in NCDS58 and at age 5 in BCS70. Whether a series of medical conditions (recurrent sore throat/ear infection, eczema, hay fever, pathological heart condition, recurrent abdominal pain) happened last year was collected at age 11 in NCDS58 and at age 10 in BCS70. A series of medical conditions (Hay fever or allergic rhinitis, recurrent vomiting or bilious attacks, dysmenorrhoea, travel sickness, recurrent abdominal pains, recurrent throat and/or ear infections requiring treatment by a doctor, severe acne, eczematous rashes, psoriasis) were recorded at age 16 in both cohorts. Medical conditions were recoded into two categories (had >=2 medical conditions versus <=1 medical conditions) |
| Wet bed | 7 in NCDS58 and 5 in BCS70 | Whether child wet bed since age 5 was asked in NCDS58 and whether child wet bed at 5 years old was asked in BCS70 |

ESM Table 5. Factor loadings for latent externalising and internalising score in NCDS58 and BCS70

solitary

miserable

worried

fearful

destructive

irritable

fights

disobedient

0.47

0.21

0.69

0.59

0.60

0.58

0.53

0.73

0.50

(one figure example of the IRT model built in Mplus)

|  | NCDS58 | | |  | BCS70 | | |
| --- | --- | --- | --- | --- | --- | --- | --- |
|  | Wave1 | Wave2 | Wave3 |  | Wave1 | Wave2 | Wave3 |
| Internalising items |  |  |  |  |  |  |  |
| Solitary | 0.21 | 0.15 | 0.30 |  | 0.33 | 0.41 | 0.45 |
| Miserable | 0.47 | 0.48 | 0.73 |  | 0.64 | 0.68 | 0.79 |
| Worried | 0.69 | 0.71 | 0.67 |  | 0.65 | 0.74 | 0.71 |
| Fearful | 0.59 | 0.59 | 0.59 |  | 0.51 | 0.62 | 0.61 |
| Externalising items |  |  |  |  |  |  |  |
| Destructive | 0.58 | 0.65 | 0.70 |  | 0.62 | 0.74 | 0.73 |
| Irritable | 0.60 | 0.62 | 0.76 |  | 0.62 | 0.70 | 0.78 |
| Fights | 0.53 | 0.52 | 0.72 |  | 0.62 | 0.71 | 0.76 |
| Disobedient | 0.73 | 0.70 | 0.68 |  | 0.75 | 0.74 | 0.76 |
| Cor (internalising, externalising) | 0.50 | 0.55 | 0.71 |  | 0.56 | 0.70 | 0.82 |

ESM Table 6. Missingness in NCDS58 (upper half) and BCS70 (lower half) across waves

|  | Mental Health | | |  | Alcohol outcomes | |
| --- | --- | --- | --- | --- | --- | --- |
| Age | Age 7 | Age 11 | Age 16 |  | Age 33 | Age 44/45 |
| Year | 1965 | 1969 | 1974 |  | 1991 | 2002/04 |
| Complete cases | 13582 | 12354 | 11000 |  | 10902 | 8953 |
| Missing due to incomplete scale* | 1468 | 2402 | 2919 |  | 140 | 60 |
| Missing due to non-participation | 1762 | 2037 | 2842 |  | 5558 | 7323 |
| Missing due to death | 821 | 840 | 872 |  | 1033 | 1297 |
| Total | 17633 | 17633 | 17633 |  | 17633 | 17633 |
| Age | Age 5 | Age 10 | Age 16 |  | Age 34 | Age 46 |
| Year | 1975 | 1980 | 1986 |  | 2004 | 2016 |
| Complete cases | 12620 | 13526 | 7871 |  | 9193 | 8265 |
| Missing due to incomplete scale | 1113 | 1230 | 3840 |  | 252 | 106 |
| Missing due to non-participation | 3250 | 2216 | 5226 |  | 7,210 | 8222 |
| Missing due to death | 585 | 596 | 631 |  | 913 | 975 |
| Total | 17568 | 17568 | 17568 |  | 17568 | 17568 |
| *Refers to those who participated in the survey but didn’t provide complete information for mental health or alcohol use | | | | | | |

ESM Text 1. Multiple imputation procedure

Multiple imputation using chained equations (MICE) was implemented to impute attrition and item non-response mainly for two reasons. First, MICE has the flexibility to model each variable based on their respective distribution and release the multivariate normality assumption needed for multivariate normal imputation. With binary outcomes and many other categorical variables as in the current study, MICE was shown to have a better performance [1]. Second, data-driven approach has shown MICE has the capability to restore representativeness of the original sample in the cohort study [2].

To increase the plausibility of Missing At Random (MAR) assumption which underlies MI and improve imputation for the missingness of the outcome, auxiliary variables were incorporated in the imputation stage, including smoke habit (Never, used to smoke, current smoker), drinking frequency (special occasion/never, 2~3 times per month, 1~3 times per week, 4+ times per week), weekly alcohol units at ages 26, 33, 42 in NCDS58 and at ages 23, 34, 42 in BCS70 [2]. These auxiliary variables were all significant predictors of later problematic drinking and thus could help improve the imputation of missing outcomes and statistical efficiency [3–5].

As missing data in multi-item instrument were best handled by imputing at item level [6,7], multi-item scales (CAGE/AUDIT-PC) were imputed at item level. To avoid convergence problems, sum score of each scale instead of item score was used as predictors in any imputation model [7]. In other words, for the imputation of non-scale variables, the sum score rather than each item was included in the imputation model, and for the imputation of each item, sum score of all the other items within the scale was included instead of each item within the scale.

To ensure compatibility between the analytical model with interaction terms and the MI model, imputation was carried out separately for the four subgroups of interest (males/females in NCDS58/BCS70) [8]. To be noticed, due to death, different target population was imputed by excluding people who died by the age of the outcome of interest.

Binary variables were imputed using logistic regression; ordered categorical variables were imputed using ordinal logistic regression; un-ordered categorical variables were imputed using multi-nominal logistic regression; continuous and normal-distributed variables were imputed using linear regression; highly skewed variables including externalising and internalising problems at all ages, and weekly alcohol units across adulthood were imputed using predictive mean match method [9].

One hundred and fifty datasets were imputed based on a two-stage calculation to ensure the precision of standard error estimates (how_many_imputations package in Stata) [10]. Distributions of complete and imputed variables were checked for abnormal imputation [11,12]. Standard MI with imputed outcome values was done in the analysis stage, as it is shown to provide robust parameter estimates in the presence of auxiliary variables associated with an incomplete outcome [5].

References

1. Zaninotto P, Sacker A. Missing data in longitudinal surveys: a comparison of performance of modern techniques. J Mod App Stat Meth. 2017 Dec 4;16[2]:378–402.

2. Mostafa T, Narayanan M, Pongiglione B, Dodgeon B, Goodman A, Silverwood RJ, et al. Improving the plausibility of the missing at random assumption in the 1958 British birth cohort: A pragmatic data driven approach. London: UCL Centre for Longitudinal Studies; 2020 p. 73. Report No.: CLS Working Paper 2020/6.

3. von Hippel PT, Lynch J. Efficiency gains from using auxiliary variables in imputation. arXiv:13115249. 2013;10.

4. White IR, Royston P, Wood AM. Multiple imputation using chained equations: Issues and guidance for practice. Statist Med. 2011 Feb 20;30[4]:377–99.

5. Sullivan TR, Salter AB, Ryan P, Lee KJ. Bias and Precision of the “Multiple Imputation, Then Deletion” method for dealing with missing outcome data. American Journal of Epidemiology. 2015 Sep 15;182[6]:528–34.

6. Eekhout I, de Vet HCW, Twisk JWR, Brand JPL, de Boer MR, Heymans MW. Missing data in a multi-item instrument were best handled by multiple imputation at the item score level. J Clin Epidemiol. 2014 Mar;67[3]:335–42.

7. Plumpton CO, Morris T, Hughes DA, White IR. Multiple imputation of multiple multi-item scales when a full imputation model is infeasible. BMC Res Notes [Internet]. 2016 Jan 26 [cited 2019 Oct 2];9. Available from: https://www.ncbi.nlm.nih.gov/pmc/articles/PMC4727289/

8. Tilling K, Williamson EJ, Spratt M, Sterne JAC, Carpenter JR. Appropriate inclusion of interactions was needed to avoid bias in multiple imputation. Journal of Clinical Epidemiology. 2016 Dec 1;80:107–15.

9. Morris TP, White IR, Royston P. Tuning multiple imputation by predictive mean matching and local residual draws. BMC Medical Research Methodology. 2014 Jun 5;14:75.

10. von Hippel PT. How many imputations do you need? A two-stage calculation using a quadratic rule. Sociological Methods & Research. 2018 Jan 18;004912411774730.

11. Eddings W, Marchenko YV. Diagnostics for multiple imputation in Stata. The Stata Journal. 2012;12[3]:353–67.

12. Marchenko YV, Eddings W. A note on how to perform multiple-imputation diagnostics in Stata. :9.

ESM Text 2. Formula illustration of statistical models*

Model 1:

Logit (PD) =α+INT1+EXT1+Sex+Cohort+C1+ε

Model 2:

Logit (PD)=α+INT1+INT2+EXT1+EXT2+Sex+Cohort+C1+C2+ε

Model 3:

Logit (PD)=α+INT1+INT2+INT3+EXT1+EXT2+EXT3+Sex+Cohort+C1+C2+C3+ε

Model 4:

Logit (PD)=α+INT1+EXT1+Sex+Cohort+Sex*Cohort+Sex*EXT1+Sex*INT1+Cohort*EXT1+Cohort*INT1+C1+ε

Model 5:

Logit (PD)=α+INT1+INT2+EXT1+EXT2+Sex+Cohort+Sex*Cohort+Sex*EXT2+Sex*INT2+Cohort*EXT2+Cohort*INT2+C1+C2+ε

Model 6:

Logit (PD)=α+INT1+INT2+INT3+EXT1+EXT2+EXT3+Sex+Cohort+Sex*Cohort+Sex*EXT3+Sex*INT3+Cohort*EXT3+Cohort*INT3+C1+C2+C3+ε

*EXT refers to externalising problems; INT refers to internalising problems; PD refers to problematic drinking (Yes/No)

ESM Text 3. A series of sensitivity analysis

Several sets of sensitivity analysis were carried out to assess the robustness of the results.

First, though latent scores are deemed to better capture the latent trait behind externalising and internalising problems and utilised in the main analysis, sum score of externalising and internalising items was calculated respectively and entered in the model to examine whether the association pattern differed from that when utilising latent externalising and internalising score.

Second, latent scores of externalising and internalising problems were modelled simultaneously to minimise confounding of unmeasured confounders, but the high correlation between them may cause the issue of potential multicollinearity and jeopardise the estimation of coefficients and standard error. Therefore, they were added into the lagged logistic regression model separately to examine potential problems of multicollinearity. In addition, a general psychological factor which captures covariation between externalising and internalising problems was derived to further articulate their respect role in each other’s relationship with problematic drinking.

Third, the endorsement on items that reflect more on problematic drinking (such as can’t stop, fail to work, cause concerns) was low in the population (ESM Table 16). As a result, compared to the full AUDIT scale, participants classified as problematic drinkers based on AUDIT-PC were mainly those who scored high either on drinking frequency or drinking quantity, which may lead to false positives of problematic drinking (ESM Table 17). Sensitivity analysis was carried out using both full and short versions of AUDIT at age 44/45 in NCDS58 to investigate potential bias due to misclassification.

Fourth, several well-established risk factors for alcohol use, such as alcohol-metabolising genes, parental alcohol use problems, peer’s drinking behaviours, were not available and thus not adjusted in the analysis. To assess the susceptibility of our results to potential unmeasured confounding factors, E-Value was calculated and reported alongside the main results. E-value evaluates the minimum strength, on risk ratio scale, that an unmeasured confounder would need to have with both exposure and the outcome in order to fully explain away the observed association, conditional on the measured confounding factors (1). The larger an E-value is, the more unmeasured confounding would be needed to explain away an effect estimate. Thus, E-value is particularly helpful in making causal inference using observational studies.

Fifth, analysis including observations with imputed outcomes may lead to bias if the model for imputing the missing outcomes was mis-specified (2). Thus, multiple imputation then deletion method was carried out as a sensitivity check (2).

1. VanderWeele TJ, Ding P. Sensitivity analysis in observational research: Introducing the E-value. Ann Intern Med. 2017 Aug 15;167(4):268.

2. von Hippel PT. Regression with missing Ys: An improved strategy for analyzing multiply imputed data. Sociological Methodology. 2007;37:83–117.

ESM Table 7 Association between standardised externalising and internalising scores and problematic drinking at age 33/34 and age 45 in two British birth cohorts^#^, OR (95% CI)

|  | PD (CAGE) at age 33/34 | | | | | |
| --- | --- | --- | --- | --- | --- | --- |
| cage6 | Model 1 | E-value | Model 2 | E-value | Model 3 | E-value |
| EXT at age 7 | 1.11 (1.05,1.16) ^***^ | 1.29 (1.18) | 1.07 (1.01,1.14) ^*^ | NA | 1.05 (0.99,1.11) | NA |
| INT at age 7 | 0.95 (0.91,1.00) | 1.19 (1.00) | 0.97 (0.92,1.02) | NA | 0.97 (0.92,1.03) | NA |
| EXT at age 11 |  |  | 1.12 (1.06,1.19) ^***^ | 1.31 (1.20) | 1.07 (1.00,1.14) ^*^ | NA |
| INT at age 11 |  |  | 0.94 (0.88,0.99) ^*^ | 1.21 (1.08) | 0.95 (0.89,1.01) | NA |
| EXT at age 16 |  |  |  |  | 1.19 (1.11,1.28) ^***^ | 1.41 (1.29) |
| INT at age 16 |  |  |  |  | 0.91 (0.84,0.99) ^*^ | 1.27 (1.08) |
| *N* | 33255 |  | 33255 |  | 33255 |  |
|  | PD (AUDIT) at age 45 | | | | | |
|  | Model 1 |  | Model 2 |  | Model 3 |  |
| EXT at age 7 | 1.07 (1.02,1.12) ^**^ | 1.22 (1.11) | 1.05 (1.00,1.10) | NA | 1.03 (0.98,1.08) | NA |
| INT at age 7 | 0.94 (0.90,0.98) ^**^ | 1.21 (1.11) | 0.96 (0.92,1.01) | NA | 0.97 (0.93,1.02) | NA |
| EXT at age 11 |  |  | 1.08 (1.03,1.13) ^**^ | 1.24 (1.14) | 1.04 (0.99,1.09) | NA |
| INT at age 11 |  |  | 0.92 (0.88,0.96) ^***^ | 1.25 (1.17) | 0.94 (0.90,0.99) ^*^ | NA |
| EXT at age 16 |  |  |  |  | 1.19 (1.12,1.27) ^***^ | 1.41 (1.31) |
| INT at age 16 |  |  |  |  | 0.87 (0.80,0.93) ^***^ | 1.35 (1.23) |
| *N* | 32842 |  | 32842 |  | 32842 |  |

^#^EXT = externalising problems; INT = internalising problems; PD = problematic drinking. Confounding factors were added chronologically as described in the method section. 30 complete datasets were imputed for this analysis.

^&^NA refers to not applicable. The value outside the bracket is E-value for the point estimate, and the value in the bracket is E-value for the limit of the confidence interval closest to the null (the strength needed to move the confidence interval to include 1).

^*^ *p* < 0.05, ^**^ *p* < 0.01, ^***^ *p* < 0.001

ESM Table 8. Interaction between externalising and internalising behaviour and sex/cohort regarding their association with problematic drinking at age 33/34 and age 45 in two British birth cohorts

|  | Main effect | P value | Interaction with sex^*^ | P value | Interaction with cohort^*^ | P value |
| --- | --- | --- | --- | --- | --- | --- |
|  | Problematic Drinking at age 33/34 (CAGE), OR (95% CI) | | | | | |
| Externalising  at age 5/7 | 1.14  (1.04,1.25) | 0.004 | 0.93  (0.85,1.02) | 0.136 | 1.02  (0.93,1.11) | 0.688 |
| Internalising  at age 5/7 | 1.01  (0.94,1.1) | 0.713 | 0.95  (0.88,1.04) | 0.268 | 0.95  (0.88,1.02) | 0.162 |
| Externalising  at age 10/11 | 1.10  (1.01,1.21) | 0.027 | 1.01  (0.93,1.10) | 0.826 | 0.98  (0.90,1.06) | 0.606 |
| Internalising  at age 10/11 | 0.98  (0.91,1.06) | 0.616 | 0.96  (0.89,1.03) | 0.256 | 1.00  (0.93,1.08) | 0.990 |
| Externalising  at age 16 | 1.06  (0.98,1.14) | 0.166 | 1.10  (1.01,1.19) | 0.028 | 0.99  (0.92,1.07) | 0.833 |
| Internalising  at age 16 | 1.09  (0.98,1.20) | 0.109 | 0.81  (0.73,0.90) | <0.001 | 0.95  (0.86,1.05) | 0.290 |
|  | Problematic Drinking at age 45 (AUDIT-PC), OR (95% CI) | | | | | |
| Externalising  at age 5/7 | 1.12  (1.05,1.20) | 0.001 | 0.9  (0.84,0.96) | 0.003 | 1.03  (0.96,1.12) | 0.379 |
| Internalising  at age 5/7 | 0.92  (0.87,0.98) | 0.007 | 1.04  (0.97,1.11) | 0.293 | 1.00  (0.94,1.07) | 0.970 |
| Externalising  at age 10/11 | 1.04  (0.97,1.11) | 0.263 | 1.00  (0.93,1.08) | 0.956 | 1.05  (0.98,1.13) | 0.163 |
| Internalising  at age 10/11 | 0.96  (0.91,1.02) | 0.183 | 0.98  (0.92,1.05) | 0.612 | 0.97  (0.91,1.03) | 0.349 |
| Externalising  at age 16 | 1.07  (1.00,1.13) | 0.037 | 1.03  (0.96,1.10) | 0.397 | 1.05  (0.98,1.11) | 0.164 |
| Internalising  at age 16 | 0.95  (0.88,1.03) | 0.211 | 0.90  (0.83,0.99) | 0.026 | 0.98  (0.9,1.07) | 0.707 |

*When exploring interaction effect, female and NCDS58 was the reference group. Confounding factors listed in ESM Table 4 were adjusted for correspondingly in each model as in the manuscript.

ESM Table 9. Association between early life externalising and internalising problems and problematic drinking at age 33/34 in two British birth cohorts^#^, OR (95% CI)

|  | Model 1 | Model 2 | Model 3 | Model 4 | Model 5 | Model 6 |
| --- | --- | --- | --- | --- | --- | --- |
| Externalising | 1.07^***^ | 1.08^**^ | 1.06^*^ |  | 1.11^***^ | 1.07^*^ |
| at age 7 | (1.03,1.11) | (1.03,1.13) | (1.00,1.11) |  | (1.06,1.16) | (1.01,1.12) |
| Internalising |  | 0.96^*^ | 0.97 | 1.01 | 0.96^*^ | 0.97 |
| at age 7 |  | (0.92,1.00) | (0.93,1.01) | (0.97,1.04) | (0.92,1.00) | (0.93,1.01) |
| Externalising |  | 1.06^**^ | 1.07^*^ |  |  | 1.09^***^ |
| at age 11 |  | (1.02,1.10) | (1.01,1.12) |  |  | (1.04,1.15) |
| Internalising |  |  | 0.95^*^ |  | 1.00 | 0.95^*^ |
| at age 11 |  |  | (0.91,0.99) |  | (0.97,1.03) | (0.91,0.99) |
| Externalising |  |  | 1.06^***^ |  |  |  |
| at age 16 |  |  | (1.03,1.10) |  |  |  |
| Internalising |  |  |  |  |  | 1.03 |
| at age 16 |  |  |  |  |  | (0.99,1.07) |
| *N* | 33255 | 33255 | 33255 | 33255 | 33255 | 33255 |

#Latent score of EXT and INT at the same age were entered into the model separately. Problematic drinking was measured using CAGE scale. Confounding factors listed in ESM Table 4 were adjusted for correspondingly in each model as in the manuscript.

^*^ *p* < 0.05, ^**^ *p* < 0.01, ^***^ *p* < 0.001

ESM Table 10. Association between early life externalising and internalising problems and problematic drinking at age 45 in two British birth cohorts^#^, OR (95% CI)

|  | Model 1 | Model 2 | Model 3 | Model 4 | Model 5 | Model 6 |
| --- | --- | --- | --- | --- | --- | --- |
| Externalising | 1.03 | 1.06^**^ | 1.04 |  | 1.07^***^ | 1.05^*^ |
| at age 7 | (1.00,1.06) | (1.02,1.11) | (1.00,1.08) |  | (1.04,1.12) | (1.01,1.09) |
| Internalising |  | 0.94^***^ | 0.96^*^ | 0.97 | 0.95^**^ | 0.96 |
| at age 7 |  | (0.91,0.97) | (0.93,1.00) | (0.95,1.00) | (0.92,0.99) | (0.93,1.00) |
| Externalising |  | 1.02 | 1.05^*^ |  |  | 1.07^***^ |
| at age 11 |  | (0.99,1.05) | (1.01,1.10) |  |  | (1.03,1.11) |
| Internalising |  |  | 0.94^***^ |  | 0.97^*^ | 0.94^***^ |
| at age 11 |  |  | (0.91,0.97) |  | (0.95,1.00) | (0.91,0.97) |
| Externalising |  |  | 1.04^**^ |  |  |  |
| at age 16 |  |  | (1.01,1.06) |  |  |  |
| Internalising |  |  |  |  |  | 0.99 |
| at age 16 |  |  |  |  |  | (0.96,1.02) |
| *N* | 32929 | 32929 | 32929 | 32929 | 32929 | 32929 |

#Latent score of EXT/INT at the same age were entered into the model separately. Problematic drinking was measured using AUDIT scale. Confounding factors listed in ESM Table 4 were adjusted for correspondingly in each model as in the manuscript.

^*^ *p* < 0.05, ^**^ *p* < 0.01, ^***^ *p* < 0.001

ESM Table 11. Comparison of associations between early life psychopathology factor^*^ and externalising and internalising problems at age 16 with problematic drinking at age 33/34 and age 45 in two British birth cohorts

|  | PD at age 33/34 (CAGE)  (n=33255) | PD at age 46 (AUDIT) (n=32929) |
| --- | --- | --- |
| Externalising problems | 1.11 (1.06, 1.16) | 1.11 (1.07, 1.15) |
| Internalising problems | 0.93 (0.87, 0.99) | 0.90 (0.86, 0.94) |
| Psychopathology factor | 1.05 (1.02, 1.09) | 1.02 (0.99, 1.05) |

*Psychopathology factor was derived using all 8 items (fights, disobedient, destructive, irritable, being worried, solitary, fearful and miserable) used to derive latent score for externalising and internalising problems. Thus, it captures the common variance of both externalising and internalising problems.

ESM Table 12. Variables collected with information about various drinking behaviours in NCDS58 and BCS70

|  |  |  |  | NCDS58 |  |  |  |  |  |  | BCS70 |  |  |  |
| --- | --- | --- | --- | --- | --- | --- | --- | --- | --- | --- | --- | --- | --- | --- |
| Year | 1981 | 1991 | 2000 | 2002/2004 | 2004 | 2008 | 2013 |  | 1996 | 2000 | 2004 | 2008 | 2012 | 2016 |
| Age | 23 | 33 | 42 | 44/46 | 46 | 50 | 55 |  | 26 | 30 | 34 | 38 | 42 | 46 |
| Drinking fre and amount |  |  |  |  |  |  |  |  |  |  |  |  |  |  |
| How often do you usually have an alcoholic drink of any kind? | √ | √ | √ | √ | √ | √ | √ |  | √ | √ | √ |  | √ | √ |
| In the last week, I have drunk no alcohol at all |  |  |  |  |  |  |  |  | √ |  |  |  |  |  |
| Since this time last week, how much shandy have you drunk? Pints (assume that one small can=half a pint) |  |  |  |  |  |  |  |  | √ |  |  |  |  |  |
| In the last seven days, not counting today, how much beer stout lager or cider have you had? | √ | √ | √ |  |  | √ |  |  |  | √ | √ |  |  |  |
| In the last seven days, how much normal strength beer stout lager stout ale or cider have you had? |  |  |  |  |  |  |  |  |  |  |  |  | √ | √ |
| In the last seven days, how much strong strength beer stout lager stout ale or cider have you had? |  |  |  |  |  |  |  |  |  |  |  |  | √ | √ |
| Since this time last week, how much beer (including lager) have you drunk? Pints |  |  |  |  |  |  |  |  | √ |  |  |  |  |  |
| In the last week, I have X pints of low alcohol beers/lagers |  |  |  |  |  |  |  |  | √ |  |  |  |  |  |
| Since this time last week, how much cider have you drunk? pints |  |  |  |  |  |  |  |  | √ |  |  |  |  |  |
| In the last week, I have X pints of Low alcohol of cider |  |  |  |  |  |  |  |  | √ |  |  |  |  |  |
| In the last 7 days, how many measures of spirits or liqueurs have you had, like gin, whisky, rum, brandy, vodka or advocat? | √ | √ | √ |  |  | √ |  |  | √ | √ | √ |  | √ | √ |
| In the last seven days, how many glasses of wine have you had? | √ | √ | √ |  |  | √ |  |  | √ | √ | √ |  |  |  |
| In the last week, I have X glasses of Low alcohol wine |  |  |  |  |  |  |  |  | √ |  |  |  |  |  |
| In the last seven days, how much wine, including sparkling wine and champagne have you had? |  |  |  |  |  |  |  |  |  |  |  |  | √ | √ |
| In the last seven days, were the glasses of wine that you drank large (250ml), standard (175ml) or small (125ml) glasses? |  |  |  |  |  |  |  |  |  |  |  |  | √ | √ |
| In the last 7 days, how many large (250ml) glasses of wine did you have? |  |  |  |  |  |  |  |  |  |  |  |  | √ | √ |
| In the last 7 days, how many standard (175ml) glasses of wine did you have? |  |  |  |  |  |  |  |  |  |  |  |  | √ | √ |
| In the last 7 days, how many standard (125ml) glasses of wine did you have? |  |  |  |  |  |  |  |  |  |  |  |  | √ | √ |
| In the last seven days, how many glasses of martini, vermouth or similar drinks have you had? | √ | √ | √ |  |  | √ |  |  | √ | √ | √ |  | √ | √ |
| In the last 7 days, how many bottles of alcopops have you had? |  |  | √ |  |  |  |  |  |  | √ | √ |  | √ | √ |
| In the last 7 days, have you had any other alcoholic drinks? |  |  | √ |  |  | √ |  |  | √ | √ | √ |  |  |  |
| As far as the amount you drink is concerned, would you say the last seven days were? | √ |  |  |  |  |  |  |  |  |  |  |  |  |  |
| In an average week, how many units do you drink? |  |  |  |  | √ |  | √ |  |  |  |  |  |  |  |
| On the days when you do drink alcohol, on average how many units do you drink in a day? |  |  |  |  | √ |  |  |  |  |  |  |  |  |  |

ESM Text 4. Conversion to UK units

Information on drinking frequency and drinking amount of different kinds of alcohol (mainly beer, wine, spirits and mixed drinks) in the past 7 days were collected across adulthood, but survey methods and questions were not identical across waves and cohorts (See ESM Table 7). To ensure comparability, weekly alcohol use at age 34 in NCDS58 and at age 33 in BCS70 were retained.

Drinks was converted into UK weekly alcohol units (AU, one unit equals 10ml or 8g of pure alcohol, which is around the average amount of alcohol one adult can process in an hour) using the following conversion: one pint of beer=two units, one pint of strong beer=three units, one pint of low alcohol beer=one unit, one glass of low alcohol wine=0.5 unit, 125ml glass of wine=one unit, 175ml glass of wine=two units, 225ml glass of wine=three units, one single measure of spirit=one unit, one glass of mixed drink=one unit. In addition, consumption of alcopops and other drinks were not calculated in both cohorts and for those who drank monthly or less, alcohol units were aligned to 0 units per week. Since alcohol units per week were highly skewed, lagged quantile regression was carried out at 25% (Q1), 50% (Q2), 75% (Q3) of the distribution.

ESM Table 13. Association between externalising and internalising behaviours and weekly alcohol units at age 33/34 in two British birth cohort^#^

|  | Model 1 | Model 2 | Model 3 |
| --- | --- | --- | --- |
| q50 |  |  |  |
| Externalising at age 7 | 0.21^**^(0.05,0.37) | 0.18^*^(0.01,0.35) | 0.13(-0.04,0.30) |
| Internalising at age 7 | -0.28^***^(-0.41,-0.15) | -0.17^*^(-0.31,-0.03) | -0.14(-0.27,0.00) |
| Externalising at age 11 |  | 0.19^*^(0.01,0.37) | 0.10(-0.08,0.28) |
| Internalising at age 11 |  | -0.26^***^(-0.40,-0.13) | -0.18^*^(-0.32,-0.04) |
| Externalising at age 16 |  |  | 0.33^***^(0.17,0.49) |
| Internalising at age 16 |  |  | -0.41^***^(-0.58,-0.24) |
| q75 |  |  |  |
| Externalising at age 7 | 0.59^***^(0.31,0.86) | 0.47^**^(0.17,0.77) | 0.34^*^(0.04,0.64) |
| Internalising at age 7 | -0.59^***^(-0.82,-0.36) | -0.42^***^(-0.66,-0.17) | -0.35^**^(-0.60,-0.10) |
| Externalising at age 11 |  | 0.52^***^(0.22,0.83) | 0.32(-0.00,0.64) |
| Internalising at age 11 |  | -0.50^***^(-0.73,-0.27) | -0.38^**^(-0.61,-0.14) |
| Externalising at age 16 |  |  | 0.71^***^(0.41,1.01) |
| Internalising at age 16 |  |  | -0.72^***^(-1.05,-0.38) |
| *N* | 33255 | 33255 | 33255 |

# Confounding factors listed in ESM Table 4 were adjusted for correspondingly in each model as in the manuscript; lagged quantile regression was conducted, and the coefficients represent changes in weekly alcohol units for one unit change in latent score of externalising and internalising problems

ESM Table 14 Distribution of AUDIT-PC score at age 45 across two cohorts (count (%))*

| Score | 0 | 1 | 2 | 3 | 4 |
| --- | --- | --- | --- | --- | --- |
|  | NCDS58 | | | | |
| Drinking frequency | 207 (4.7) | 436 (9.8) | 907 (20.4) | 1481 (33.4) | 1408 (31.7) |
| Drinking quantity | 1769 (39.8) | 1188 (26.8) | 788 (17.8) | 410 (9.2) | 285 (6.4) |
| Can’t stop | 4081 (91.9) | 167 (3.8) | 76 (1.7) | 93 (2.1) | 23 (0.5) |
| Fail to work | 4100 (92.3) | 284 (6.4) | 31 (0.7) | 22 (0.5) | 3 (0.1) |
| Cause concern | 3936 (88.7) |  | 241 (5.4) |  | 263 (5.9) |
|  | BCS70 | | | | |
| Drinking frequency | 385 (9.6) | 596 (14.9) | 924 (23.1) | 1314 (32.9) | 775 (19.4) |
| Drinking quantity | 1881 (47.2) | 1205 (30.3) | 602 (15.1) | 201 (5.1) | 93 (2.3) |
| Can’t stop | 3447 (86.4) | 313 (7.9) | 102 (2.6) | 79 (2.0) | 47 (1.2) |
| Fail to work | 3632 (91) | 288 (7.2) | 36 (0.9) | 18 (0.5) | 16 (0.4) |
| Cause concern | 3481 (87.2) |  | 246 (6.2) |  | 267 (6.7) |

ESM Table 15 Cross-tabulation of problematic drinking defined by AUDIT and AUDIT-PC scale across sex in NCDS58

|  | AUDIT-PC | | | |
| --- | --- | --- | --- | --- |
|  | Male | | Female | |
|  | Yes | No | Yes | No |
| AUDIT |  |  |  |  |
| Yes | 1534 (96.7) | 53 (3.3) | 607 (93.2) | 44 (6.8) |
| No | 399 (14.0) | 2453 (86.0) | 294 (7.6) | 3569 (92.4) |

ESM Table 16. Association between early life externalising and internalising problems and problematic drinking (PD) at age 45 measured using different AUDIT scale in NCDS58, OR (95%CI)

|  | PD at age 46 (full AUDIT) | | | PD at age 46 (AUDIT-PC) | | |
| --- | --- | --- | --- | --- | --- | --- |
|  | Model 1 | Model 2 | Model 3 | Model 1 | Model 2 | Model 3 |
| Externalising | 1.06^*^ | 1.04 | 1.02 | 1.05^*^ | 1.04 | 1.02 |
| at age 7 | (1.00,1.11) | (0.98,1.10) | (0.96,1.08) | (1.00,1.10) | (0.99,1.09) | (0.97,1.08) |
| Internalising | 0.96^*^ | 0.98 | 0.99 | 0.94^**^ | 0.96 | 0.97 |
| at age 7 | (0.92,0.99) | (0.94,1.02) | (0.95,1.04) | (0.90,0.98) | (0.91,1.00) | (0.92,1.01) |
| Externalising |  | 1.07^*^ | 1.04 |  | 1.05 | 1.02 |
| at age 11 |  | (1.01,1.14) | (0.98,1.11) |  | (0.99,1.11) | (0.96,1.09) |
| Internalising |  | 0.93^**^ | 0.95 |  | 0.96 | 0.97 |
| at age 11 |  | (0.89,0.98) | (0.91,1.00) |  | (0.91,1.00) | (0.93,1.02) |
| Externalising |  |  | 1.10^***^ |  |  | 1.08^**^ |
| at age 16 |  |  | (1.04,1.16) |  |  | (1.03,1.14) |
| Internalising |  |  | 0.88^***^ |  |  | 0.90^**^ |
| at age 16 |  |  | (0.83,0.94) |  |  | (0.85,0.96) |
| *N* | 16336 | 16336 | 16336 | 16336 | 16336 | 16336 |

^*^ *p* < 0.05, ^**^ *p* < 0.01, ^***^ *p* < 0.001; AUDIT-PC is short for the primary version of the AUDIT scale. Confounding factors listed in ESM Table 4 were adjusted for correspondingly in each model as in the manuscript. 30 complete datasets were imputed for this analysis.

ESM Table 17. Association between sum score of early life externalising and internalising problems and problematic drinking (PD) at age 33/34 and age 45 in two British birth cohorts^#^, OR (95% CI)

|  | PD at age 33/34 (CAGE) | | | PD at age 45 (AUDIT-PC) | | |
| --- | --- | --- | --- | --- | --- | --- |
|  | Model 1 | Model 2 | Model 3 | Model 1 | Model 2 | Model 3 |
| Externalising | 1.23^***^ | 1.16^*^ | 1.11 | 1.15^**^ | 1.11^*^ | 1.08 |
| at age 7 | (1.11,1.36) | (1.03,1.30) | (0.99,1.25) | (1.05,1.25) | (1.01,1.23) | (0.98,1.20) |
| Internalising | 0.91 | 0.94 | 0.95 | 0.84^***^ | 0.89^*^ | 0.92 |
| at age 7 | (0.81,1.02) | (0.83,1.06) | (0.84,1.07) | (0.77,0.91) | (0.81,0.98) | (0.83,1.01) |
| Externalising |  | 1.24^**^ | 1.11 |  | 1.16^**^ | 1.08 |
| at age 11 |  | (1.09,1.41) | (0.97,1.28) |  | (1.05,1.28) | (0.97,1.20) |
| Internalising |  | 0.92 | 0.94 |  | 0.85^***^ | 0.89^*^ |
| at age 11 |  | (0.84,1.00) | (0.85,1.04) |  | (0.78,0.92) | (0.82,0.97) |
| Externalising |  |  | 1.48^***^ |  |  | 1.36^***^ |
| at age 16 |  |  | (1.26,1.74) |  |  | (1.20,1.54) |
| Internalising |  |  | 0.85^*^ |  |  | 0.76^***^ |
| at age 16 |  |  | (0.75,0.98) |  |  | (0.69,0.85) |
| *N* | 33255 | 33255 | 33255 | 32929 | 32929 | 32929 |

# Externalising and internalising problems were measured using the sum score of corresponding items. Confounding factors listed in ESM Table 4 were adjusted for correspondingly in each model as in the manuscript. 30 complete datasets were imputed for this analysis.

^*^ *p* < 0.05, ^**^ *p* < 0.01, ^***^ *p* < 0.001

ESM Table 18. Comparison of the coefficients before and after adjusting previous externalising and internalising problems*, OR (95% CI)

|  | Problematic Drinking (CAGE)  at age 33/34 | | Problematic Drinking (AUDIT-PC)  at age 45 | |
| --- | --- | --- | --- | --- |
|  | Not adjusted | Adjusted | Not adjusted | Adjusted |
| Externalising  at age 5/7 | 1.10  (1.05,1.15) | NA | 1.06  (1.03,1.10) | NA |
| Internalising  at age 5/7 | 0.96  (0.92,1.00) | NA | 0.94  (0.91,0.98) | NA |
| Externalising  at age 10/11 | 1.12  (1.07,1.17) | 1.09  (1.04,1.15) | 1.09  (1.05,1.13) | 1.07  (1.03,1.11) |
| Internalising  at age 10/11 | 0.95  (0.91,0.98) | 0.95  (0.92,0.99) | 0.93  (0.90,0.96) | 0.94  (0.91,0.97) |
| Externalising  at age 16 | 1.14  (1.09,1.19) | 1.11  (1.06,1.16) | 1.12  (1.09,1.16) | 1.11  (1.07,1.15) |
| Internalising  at age 16 | 0.91  (0.86,0.96) | 0.93  (0.87,0.99) | 0.88  (0.84,0.91) | 0.90  (0.86,0.94) |

ESM Table 19. Association between early life externalising and internalising problems and problematic drinking (PD) at age 33/34 and age 45 in two British birth cohorts^#^, OR (95% CI)

|  | PD at age 33/34 (CAGE) | | | PD at age 45 (AUDIT-PC) | | |
| --- | --- | --- | --- | --- | --- | --- |
|  | Model 1 | Model 2 | Model 3 | Model 1 | Model 2 | Model 3 |
| Externalising | 1.08^***^ | 1.05^*^ | 1.04 | 1.08^***^ | 1.06^*^ | 1.04 |
| at age 7 | (1.03,1.14) | (1.00,1.11) | (0.98,1.09) | (1.03,1.12) | (1.01,1.11) | (1.00,1.09) |
| Internalising | 0.96 | 0.97 | 0.97 | 0.93^***^ | 0.95^*^ | 0.96 |
| at age 7 | (0.92,1.00) | (0.93,1.02) | (0.93,1.02) | (0.90,0.97) | (0.91,0.99) | (0.92,1.00) |
| Externalising |  | 1.10^***^ | 1.06^*^ |  | 1.07^**^ | 1.04 |
| at age 11 |  | (1.04,1.15) | (1.00,1.12) |  | (1.02,1.12) | (0.99,1.08) |
| Internalising |  | 0.95^*^ | 0.96 |  | 0.94^**^ | 0.96^*^ |
| at age 11 |  | (0.91,1.00) | (0.92,1.01) |  | (0.91,0.98) | (0.92,1.00) |
| Externalising |  |  | 1.10^***^ |  |  | 1.11^***^ |
| at age 16 |  |  | (1.05,1.16) |  |  | (1.06,1.15) |
| Internalising |  |  | 0.94^*^ |  |  | 0.90^***^ |
| at age 16 |  |  | (0.88,1.00) |  |  | (0.85,0.95) |
| *N* | 20095 | 20095 | 20095 | 17218 | 17218 | 17218 |

# Only cases with complete outcome were retained in the analysis. Problematic drinking was measured using CAGE scale at age 33/34 and AUDIT-PC scale at age 45. Confounding factors listed in ESM Table 4 were adjusted for correspondingly in each model as in the manuscript.

^*^ *p* < 0.05, ^**^ *p* < 0.01, ^***^ *p* < 0.001
